# NIR-responsive carrier-free nanoparticles based on berberine hydrochloride and indocyanine green for synergistic antibacterial therapy and promoting infected wound healing

**DOI:** 10.1093/rb/rbad076

**Published:** 2023-08-30

**Authors:** Youyu Duan, Peiyao Xu, Panyuan Ge, Linfei Chen, Ying Chen, Ranjith Kumar Kankala, Shibin Wang, Aizheng Chen

**Affiliations:** Institute of Biomaterials and Tissue Engineering, Huaqiao University, Xiamen, Fujian 361021, PR China; Fujian Provincial Key Laboratory of Biochemical Technology, Huaqiao University, Xiamen, Fujian 361021, PR China; Institute of Biomaterials and Tissue Engineering, Huaqiao University, Xiamen, Fujian 361021, PR China; Fujian Provincial Key Laboratory of Biochemical Technology, Huaqiao University, Xiamen, Fujian 361021, PR China; Institute of Biomaterials and Tissue Engineering, Huaqiao University, Xiamen, Fujian 361021, PR China; Fujian Provincial Key Laboratory of Biochemical Technology, Huaqiao University, Xiamen, Fujian 361021, PR China; Institute of Biomaterials and Tissue Engineering, Huaqiao University, Xiamen, Fujian 361021, PR China; Fujian Provincial Key Laboratory of Biochemical Technology, Huaqiao University, Xiamen, Fujian 361021, PR China; Institute of Biomaterials and Tissue Engineering, Huaqiao University, Xiamen, Fujian 361021, PR China; Fujian Provincial Key Laboratory of Biochemical Technology, Huaqiao University, Xiamen, Fujian 361021, PR China; Institute of Biomaterials and Tissue Engineering, Huaqiao University, Xiamen, Fujian 361021, PR China; Fujian Provincial Key Laboratory of Biochemical Technology, Huaqiao University, Xiamen, Fujian 361021, PR China; Institute of Biomaterials and Tissue Engineering, Huaqiao University, Xiamen, Fujian 361021, PR China; Fujian Provincial Key Laboratory of Biochemical Technology, Huaqiao University, Xiamen, Fujian 361021, PR China; Institute of Biomaterials and Tissue Engineering, Huaqiao University, Xiamen, Fujian 361021, PR China; Fujian Provincial Key Laboratory of Biochemical Technology, Huaqiao University, Xiamen, Fujian 361021, PR China

**Keywords:** phytochemicals, photothermal therapy, antibacterial, wound healing

## Abstract

Bacterial infections cause severe health conditions, resulting in a significant economic burden for the public health system. Although natural phytochemicals are considered promising anti-bacterial agents, they suffer from several limitations, such as poor water solubility and low bioavailability *in vivo*, severely restricting their wide application. Herein, we constructed a near-infrared (NIR)-responsive carrier-free berberine hydrochloride (BH, phytochemicals)/indocyanine green (ICG, photosensitizer) nanoparticles (BI NPs) for synergistic antibacterial of an infected wound. Through electrostatic interaction and π–π stacking, the hydrophobic BH and amphiphilic ICG are initially self-assembled to generate carrier-free nanoparticles. The obtained BI NPs demonstrated NIR-responsive drug release behavior and better photothermal conversion efficiency of up to 36%. In addition, BI NPs stimulated by NIR laser exhibited remarkable antibacterial activity, which realized the synergistic antibacterial treatment and promoted infected wound healing. In summary, the current research results provided a candidate strategy for self-assembling new BI NPs to treat bacterial infections synergistically.

## Graphical Abstract

**Figure:**
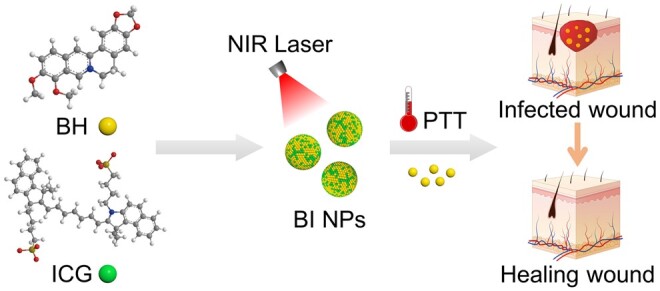

## Introduction

Bacterial infection poses a significant threat to skin wound healing [1], which prolongs the healing time and leads to complications such as bacteremia [2] and even death in severe cases [3]. The emergence of antibiotics has effectively alleviated this phenomenon. Nevertheless, bacterial resistance has been produced by the improper use of antibiotics, which has caused a significant economic burden on global public health [4]. To solve this global problem, it is required to develop an antibacterial material that could reduce the risk of bacterial resistance and effectively kill bacteria to replace traditional antibiotics.

Phytochemicals refer to an extensive range of chemical compounds found in plants. Based on their chemical structure, these phytochemicals are classified into several major categories: alkaloids, flavonoids, terpenoids, polyphenols and sulfur-containing phytochemicals [5]. These phytochemicals have attracted significant attention from researchers towards various physiological functions, such as anti-cancer, anti-microbial, anti-oxidation, anti-thrombosis, regulation of immune function and inhibition of inflammatory processes [6]. Many studies indicated that phytochemicals showed a significant antibacterial effect and low drug resistance [7, 8]. Berberine hydrochloride (BH) is a natural alkaloid with low toxicity extracted from traditional Chinese medicines (for instance, Rhizoma Coptidis), possessing antiviral, antibacterial, anti-inflammatory and anti-tumor activities [9–11]. BH has a broad spectrum of antibacterial activity and exerts antibacterial effects on bacteria through multiple mechanisms, such as reducing the activity of topoisomerase I/II to affect bacterial DNA synthesis, reducing the number of flagella on the surface of bacteria to impact bacterial adhesion and inhibiting the synthesis of proteins and lipids to interfere with bacterial reproduction [12, 13]. For *Staphylococcus aureus (S. aureus)*, BH exerts an antibacterial effect by inhibiting the activity of glutamine synthetase and transpeptidase affect the synthesis of amino acids and the pathogenicity of virulence factors [14]. It is worth noting that the inhibitory effect of BH on Gram-negative (G-) bacteria is much lower than that on Gram-positive (G+) bacteria, but the inhibitory effect on Shigella (G-) is very significant [15–17]. Therefore, BH has been formulated into tablets for treating gastrointestinal diseases clinically, such as gastroenteritis and bacillary dysentery [18]. However, poor water solubility, low bioavailability and easy metabolic clearance *in vivo* have hindered the broad application of BH.

In recent decades, with the continuous improvement in nanotechnology, various drug delivery systems based on nanoplatforms have emerged as an effective approach to overcome the shortcomings of clinical applications. Drug delivery systems could achieve controlled release of drugs at specific sites, enhance the endurance and water solubility of drugs. Researchers came up with several nano-drug delivery systems, such as liposomes [19], polymers [20], metal-organic frameworks (MOFs) [21] and proteins [22], which could effectively promote drug absorption and on-demand localized release [23]. Nevertheless, carrier-assisted nanomedicines have limited capacity to load drugs [24], usually <10% [25]. Moreover, the carrier-induced side effects [26] may hinder their application process. Therefore, carrier-free nanomaterials without or with minimal use of inert materials have attracted significant interest from researchers [27–29]. The carrier-free nanomaterials are completely self-assembled by small molecules with pharmacological activity through π–π stacking, hydrophobic interactions, electrostatic force or hydrogen bonding [30, 31], which avoids the problem of biosafety of nanocarriers to be investigated and achieves high drug loading. In addition, this preparation method is simple, green and environmentally friendly. Previous studies indicated that BH could form nanostructures with other phytochemicals such as flavonoid glycosides [32], rhein [33], cinnamic acid [34], tannic acid [35] and rhamnolipid [36] through intermolecular forces, which could effectively improve antibacterial activity and anti-inflammatory effects. However, the uncontrolled and incomplete drug release of these phytochemical nanoparticles has limited their therapeutic effect in practical applications.

In recent years, near-infrared (NIR)-responsive drug release systems received considerable attention due to their straightforward operation, simple control and effective tissue penetration. Briefly, once an NIR laser is irradiated, drug release is achieved by the photothermal effect, which refers to the increase in temperature caused by the photosensitizer [37]. Furthermore, this temperature rise also induces bacteria death, which acts synergically with drugs and achieves higher antibacterial effect. Photothermal therapy (PTT) is a prospective treatment for bacteria and bacterial biofilms [38, 39]. It has been reported that combining PTT and natural small molecules with antibacterial activity could promote drug penetration and inhibit bacterial growth [40]. Indocyanine green (ICG, an NIR fluorescent dye) is only approved for clinical imaging (cancer diagnosis), which also has the potential to be used for PTT [41]. Due to the two polyaromatic polyene groups (hydrophobic) and two sulfonate groups (hydrophilic) of the ICG molecule, it could be combined with other drugs such as methotrexate [42], cisplatin [43] and doxorubicin [44] to construct NIR-responsive drug release systems.

**Figure 1.**
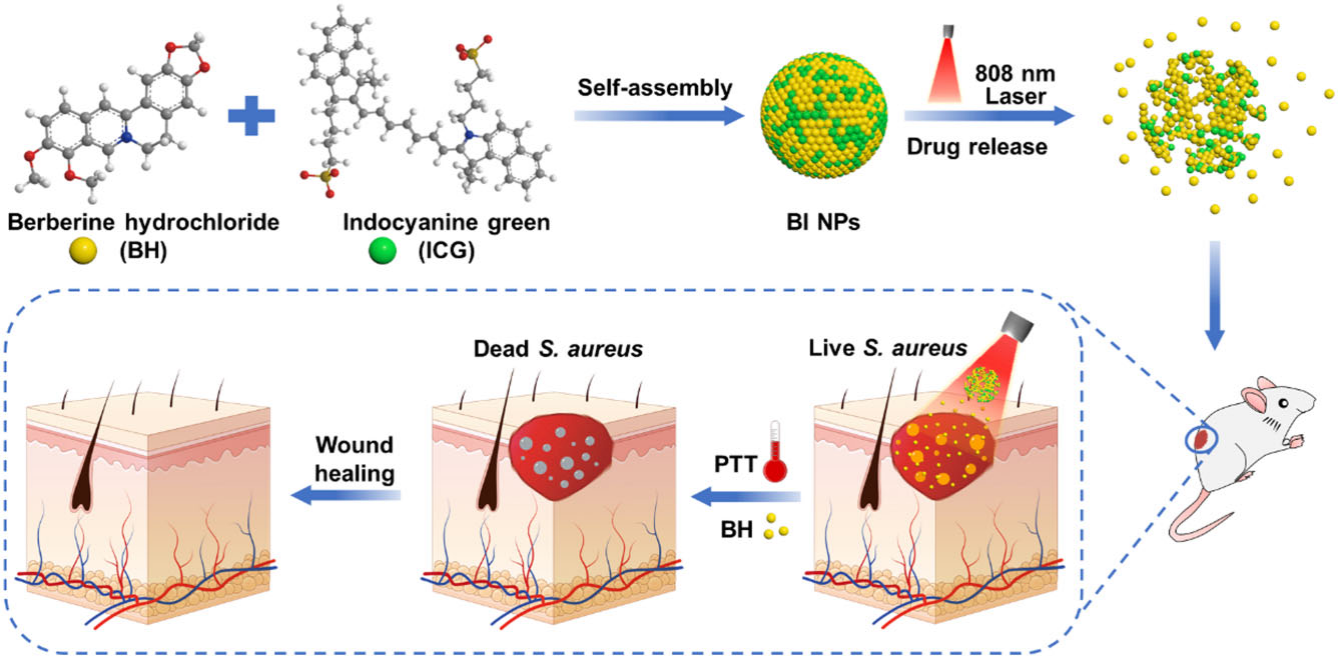
Schematic diagram showing the preparation of carrier-free BI NPs and the promotion of *S.aureus*-infection wound healing.

Herein, we constructed an NIR-responsive antibacterial platform based on BH/ICG nanoparticles (BI NPs) by direct self-assembly for synergistic chemotherapy/PTT (Figure 1). Firstly, due to electrostatic interaction and π–π stacking, BH (hydrophobic) and ICG (amphipathic) were self-assembled into BI NPs with homogeneous dispersion, excellent stability and good biocompatibility. Under an NIR laser radiation, the photothermal effect was triggered, which destroyed the nanostructure of BI NPs to release BH. In addition, it damaged the bacterial cell membrane to promote BH in the bacteria. Furthermore, the improved antibacterial activity of BI NPs was also demonstrated *in vitro*. Finally, BI NPs were successfully applied to repair the *S.aureus*-infected skin wounds.

## Materials and methods

### Materials

BH was attained from J&K Scientific (Beijing, China). ICG was attained from Sangon Biotech. Co., Ltd. (Shanghai, China). Dimethyl sulfoxide (DMSO) and agar were attained from Sinopharm United Medical Device Co., Ltd. (Beijing, China). Luria-Bertani (LB) was attained from Qingdao Hope Bio-Technology Co., Ltd. (Shandong, China). *S. aureus* (ATCC 29213) and *Escherichia coli* (*E. coli*, ATCC 25922) were attained from the China Center of Industrial Culture Collection (Beijing, China).

### Preparation of BI NPs

A facile self-assembly approach was used to prepare BI NPs, with minor adjustments based on the reported conditions [32–34]. Firstly, 5 ml of DMSO was used to dissolve BH and ICG, followed by 2 h of stirring. Secondly, deionized water was utilized to dialyze the mixture of solution (MWCO = 10 kDa). Every 3 h, deionized water was changed. Then, to obtain the nanoparticles, the resulting dispersion was centrifuged (12 000 rpm, 10 min).

### Characterizations of BI NPs

The morphology and size of BI NPs were investigated using field emission-scanning electron microscopy (FE-SEM, SU5000, Hitachi, Tokyo, Japan) and transmission electron microscopy (TEM, Thermo Fisher Talos F200X, MA, USA). Ultraviolet-visible-Near Infrared (UV-vis-NIR) spectrophotometer (UV-1800, Mapada, Shanghai, China) was used to measure the UV absorption spectra of the samples, with a scanning range of 200–1100 nm. An attenuated total internal reflectance Fourier-transform infrared (ATR-FTIR) spectrophotometer (Nicolet iS50, Thermo Fisher Scientific, Waltham, MA, USA) was employed to measure the infrared absorption spectra of samples. The surface potential of BI NPs dispersed in deionized water was evaluated using the dynamic light scattering measurements (NanoBrook Omni, Brookhaven, New York, USA). An X-ray diffraction analyzer (XRD, SmartLa, Rigaku, Tokyo, Japan) was utilized to determine the crystal structure of the sample.

### Drug release study

In order to explore the effect of NIR laser on BH release behavior from BI NPs, 1 ml of BI NPs dispersion (1 mg/ml) was incubated in a shaker at 37°C. As for NIR groups, an 808 nm NIR laser was used to irradiate BI NPs dispersion for 3 min. After that, BI NPs dispersion was centrifuged and 1 ml of supernatant was taken at each appointed time (0.5, 1, 2, 4, 6, 8, 12, 24, 48, 60, 72, 84, 96, 120, 144 and 168 h), and 1 ml supernatant was taken to measure absorbance value at 360 nm using a UV-vis spectrophotometer. In addition, an equal amount of phosphate buffered saline (PBS) should be added immediately after each removal of the supernatant. The concentrations of BH were calculated against the matching standard curve at each time point. The cumulative release was calculated as described in Supplementary data.

### Photothermal property analysis

To assess the photothermal performance of BI NPs, the photothermal effects of several samples under NIR laser irradiation was initially compared. An 808 nm NIR laser (1.0 W/cm^2^) was used to irradiate 200 μl of water, ICG solution and BI NPs dispersion. Next, the photothermal property of BI NPs was then examined in relation to the impacts of power density and concentration. An NIR thermal imaging instrument (H16, Hikvision, Hangzhou, China) was employed to track all temperature changes every 30 s during the process. The photothermal stability of BI NPs was investigated by repeated irradiation for four times under the same conditions. The photothermal conversion efficiency (η) was calculated according to the previously reported method [45], and the detailed calculation procedure is shown in Supplementary data.

### Assessment of *in vitro* antibacterial activity

The antibacterial effects of BI NPs on *S.aureus* and *E.coli in vitro* were investigated using the plate counting method. Firstly, the bacteria were inoculated in LB and cultured in a shaker (12 h, 37°C, 150 rpm). Then, equal amounts of bacteria suspension (100 μl, 10^7^ CFU/ml) were mixed with different concentrations of BH, ICG solution and BI NPs dispersion, respectively, and constantly shaken at 37°C for 18 h. Before shaking, the NIR groups were additionally exposed to an 808 nm NIR laser (1.0 W/cm^2^) for 3 min. Subsequently, the treated bacterial samples were then diluted appropriate times and evenly spread over LB agar plates for another 18 h of incubation at 37°C. Finally, the colonies formed were photographed and counted to calculate the inhibition rate.

### Morphological observations

A live/dead bacterial viability kit was utilized to stain the bacterial after treatment with different samples and culture at 37°C for 18 h. Then, a fluorescence microscopy (Axio Observer 3, Zeiss, Oberkochen, Germany) was utilized to capture the fluorescent images of bacteria.

The bacterial-specific morphological alterations were examined using the FE-SEM. The bacterial suspension was treated as described above and fixed with glutaraldehyde (2.5%) for 4–6 h. Next, the bacteria were dehydrated by gradients of 20%, 40%, 60%, 80% and 100% ethanol. Finally, the bacterial suspension after the last dehydration was diluted with ethanol to a suitable concentration and then dropped on the silicon wafer. After naturally drying, the bacteria were observed with FE-SEM.

### Evaluation of *in vivo* antibacterial effectiveness

All animal experiments in our work were performed under the guideline approved by the Institutional Animal Care and Use Committee of Huaqiao University and following the Administration of Affairs Concerning Experimental Animals of China. Female ICR mice (average body weight: 30 g) were randomly allocated into six groups: (i) control, (ii) BH, (iii) ICG, (iv) BI NPs, (v) ICG + NIR and (vi) BI NPs + NIR (n = 3). To establish the wound infection model, a dorsal skin wound (d = 6 mm) was created in each mouse and subsequently infected with *S.aureus* suspension for 1 day. The infected wounds were treated with 50 μl of different samples carefully. The wound areas in the NIR group required an additional 3 min of exposure with the 808 nm NIR laser (1.0 W/cm^2^). Subsequently, the wound areas were photographed and measured every 2 days. The formula below was used to determine the relative wound area:

$$\mathrm{Relative}\;\mathrm{wound}\;\mathrm{area}\;\left(\%\right)=\frac{S_{n}}{S_{0}}\times 100$$

$S_{0}$ and $S_{n}$ refer to the wound area on Day 0 and Day n, respectively.

For analyzing the antibacterial activity of BI NPs *in vivo*, the exudate of the infected wound was taken every 2 days. The bacterial suspension was diluted with PBS before being placed on LB agar plates for incubation (18 h, 37°C). Then, the colonies were formed and photographed.

The mice were euthanized on the ninth day, and the wounded tissues were gathered and stored with paraformaldehyde (4%). Then, they were examined using hematoxylin and eosin (H&E) staining and Masson’s trichrome staining and an optical microscope was used to capture the histological images.

### Biocompatibility

To evaluate the cytocompatibility of BI NPs, the mouse fibroblast cells (L929) were treated separately with different concentrations of BI NPs dispersion for 24 h. After that, the cell viability of each group was measured using Cell Counting Kit-8 (CCK-8), and the cell survival rate was calculated following the formula:

$$\mathrm{Cell}\;\mathrm{survival}\;\left(\%\right)=\frac{{\mathrm{OD}}_{\mathrm{sample}}}{{\mathrm{OD}}_{\mathrm{control}}}\times 100$$

The hemocompatibility was measured by fresh mouse blood. One milliliter of blood was centrifuged (10 min, 1500 rpm, 4°C) and washed with PBS. Then, the erythrocyte suspension was diluted to 5% (v/v) and incubated with different concentrations of BI NPs dispersion at 37°C for 2 h. Next, the mixture was centrifuged to collect the supernatant, and its absorbance at 540 nm was determined. The hemolysis was determined following the formula:

$$\mathrm{Hemolysis}\;\left(\%\right)=\frac{{\mathrm{OD}}_{\mathrm{sample}}-{\mathrm{OD}}_{\mathrm{PBS}}}{{\mathrm{OD}}_{\mathrm{water}}-{\mathrm{OD}}_{\mathrm{sample}}}\times 100$$

To assess the biocompatibility of different samples *in vivo*, the major organs of mice were stained with H&E staining and photographed with an optical microscope.

## Results and discussion

### Preparation and characterization of BI NPs

BI NPs were obtained by self-assembling BH and ICG in an aqueous solution. The original morphology of unprocessed BH and ICG were irregular block crystals (see Supplementary Figure S1). The mass ratio of drugs significantly affects the formation process of self-assembled particles [46]. To produce BI NPs with optimal morphology, the impact of drug mass ratio on self-assembled particles were examined (see Supplementary Figure S2). The critical mass ratio for the formation of apparent nanoparticles was 1:0.7. At the reduced mass of ICG, no obvious dispersed nanoparticles were formed. With increased ICG feeding quality, the obtained nanoparticles showed better sphericity and smaller size. However, when the mass ratio was higher than 1:1, the addition of ICG showed no discernible impact on the morphology and size of nanoparticles. The combination of BH and ICG possibly reached saturation at the mass ratio of 1:1. Therefore, the mass ratio of BH to ICG was decided upon as 1: 1 in subsequent experiments from an economic standpoint.

**Figure 2.**
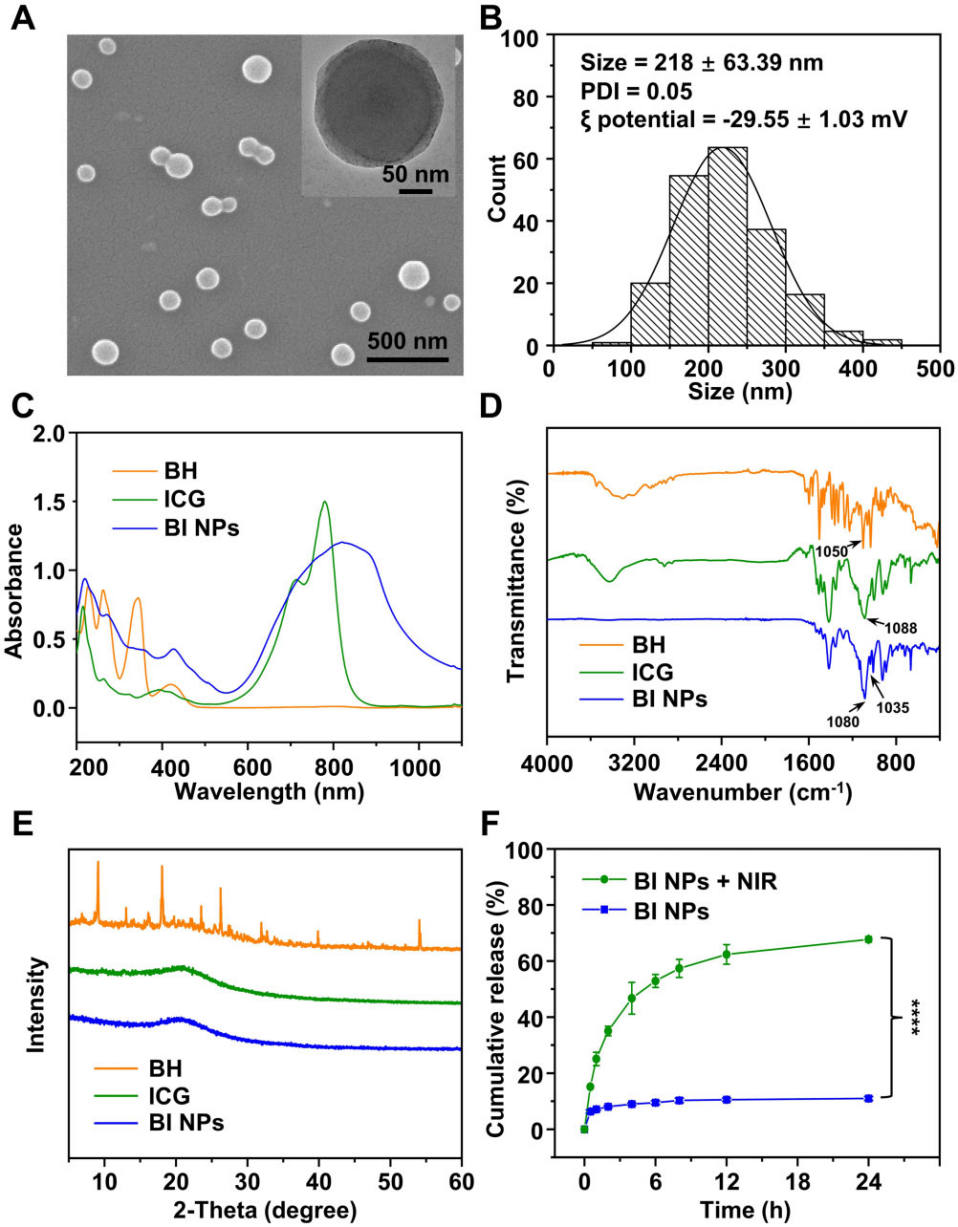
Characteristics of BI NPs. (**A**) SEM image (inset: TEM image) and (**B**) size distribution of BI NPs. (**C**) UV-vis-NIR absorbance spectra, (**D**) FT-IR spectra, (**E**) XRD of BH, ICG and BI NPs. (**F**) Release curves of BH from BI NPs exposed to the NIR in PBS, ****P < 0.001 (*n* = 3).

SEM and TEM were used to characterize the microstructure of BI NPs (Figure 2A), BI NPs showed apparent core-shell structure, uniform particle size and outstanding dispersion. Figure 2B depicted the particle size distribution of BI NPs, which had an average particle size of approximately 208.26 ± 46.09 nm with a polydispersity index of 0.05. Furthermore, BI NPs had a surface charge of −29.55 ± 1.03 mV in water.

The structural characteristics of BI NPs were studied by UV-vis-NIR, ATR-FTIR, and XRD analyses. The UV-vis-NIR spectrometer results (Figure 2C) showed that BI NPs had a widened and shorter characteristic absorption peak of ICG (780 nm) and a distinctive absorption peak of BH (360 nm), indicating the successful formation of BI NPs. The characteristic absorption peak of ICG was redshifted from 780 to 820 nm, which indicated that π–π stacking may occur between molecules. Furthermore, the coexistence of BH and ICG in the system was demonstrated by the distinctive absorption peaks of BH (1050 cm^−1^, the aromatic alkyl group) and ICG (1088 cm^−1^, the sulfonic acid group) in the FT-IR spectrum of BI NPs (Figure 2D). In addition, in the absorption spectrum of BI NPs, the absorption peak of ICG (sulfonate group) was redshifted from 1088 cm^−1^ to 1080 cm^−1^, indicating that the electrostatic interaction may have been formed between the sulfonate group of ICG and the quaternary ammonium ion of BH [46]. These results showed that the interaction between BH and ICG molecules may occur through π–π stacking and electrostatic interactions, resulting in the self-assembly to form BI NPs.

The X-ray diffractograms of BH, ICG and BI NPs were shown in Figure 2E. The unprocessed ICG had no prominent peak type to indicate an amorphous crystal structure. The unprocessed BH displayed distinct crystallographic peaks at 2θ of 9.06°, 18.1°, 23.58° and 54.06°, respectively. All of the BH-related diffraction peaks in BI NPs disappeared compared to BH, leaving only one broad peak with an amorphous crystal structure, partially supporting BI NPs.

The BH release behavior of BI NPs was investigated by the direct method. According to the experimental findings displayed in Figure 2F, the release rate of BH increased significantly after NIR laser irradiation compared with the control group. After 24 h, the BI NPs + NIR group’s cumulative BH release rate was 67.73%, considerably greater than the BI NPs group’s rate (10.98%). This might be due to the interaction between BH and ICG molecules being disrupted by laser irradiation. Therefore, under the trigger of the NIR laser, BI NPs could be employed to ensure precise and effective BH release at the therapeutic site.

**Figure 3.**
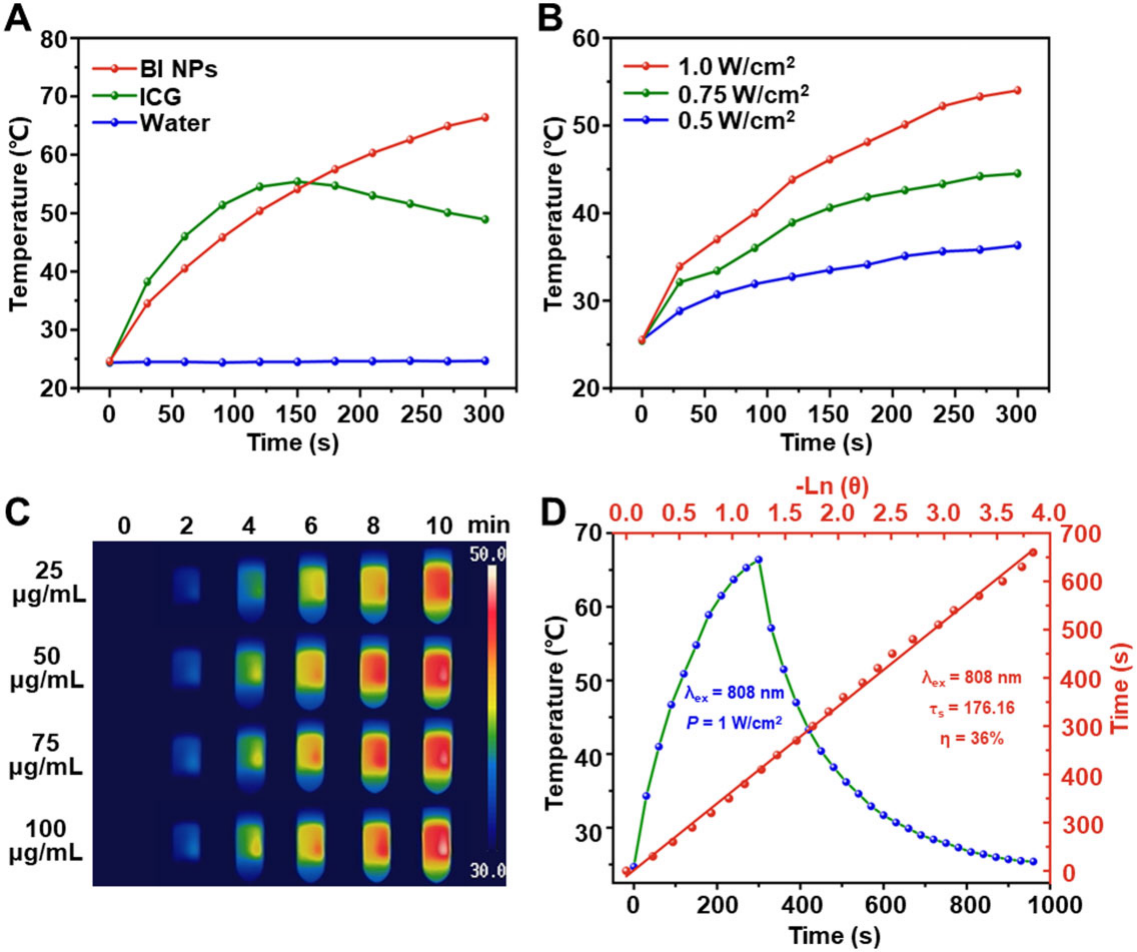
*In vitro* photothermal performance of BI NPs. Temperature change curves of (**A**) different samples (BI NPs, ICG and water) at the same laser power densities (1.0 W/cm^2^) and (**B**) BI NPs dispersion (50 μg/ml) under different laser power densities. (**C**) Infrared imaging of BI NPs dispersion in different concentrations (laser power density: 0.75 W/cm^2^). (**D**) Photothermal performance of BI NPs with linear analysis.

The photothermal heating effects of several groups of samples were evaluated to investigate the photothermal effect of BI NPs (Figure 3A). After irradiation with an NIR laser, the maximum temperatures (T_max_) of BI NPs dispersion and ICG solution were 66.4°C and 48.9°C, respectively. Notably, under the same irradiation conditions, the T_max_ of BI NPs dispersion was higher than ICG solution, which could be due to the addition of BH showing no influence on the photothermal effect of ICG. Furthermore, the stable wrapping of ICG in the self-assembled nanoparticles also reduced its interaction with free radicals/ions in the solvent. Figure 3B and 3C demonstrated the heating process of BI NPs dispersion as a function of laser power density and concentration, separately. According to Figure 3B, at the same concentration of BI NPs dispersion (50 μg/ml), T_max_ increased from 36.3°C (0.5 W/cm^2^) to 44.5°C (0.75 W/cm^2^) and 54.0°C (1.0 W/cm^2^) as the laser power density increased. The results showed that the increase in temperature was positively correlated with the laser power density. Different concentrations of BI NPs dispersion were irradiated with an NIR laser (0.75 W/cm^2^), and the photothermal conversion effects were recorded by an infrared imaging camera (Figure 3C). As the concentration of BI NPs increased, the rate of heating rose as well, ultimately improving the T_max_. The results shown the photothermal conversion capacity of BI NPs dispersion also enhanced with the increasing concentration.

To further explore the photothermal stability, the BI NPs dispersion was subjected to four cycles of excitation using the same optical power density. As depicted in Supplementary Figure S3, BI NPs dispersion could be heated up to 41.9°C at the first excitation and could still be heated up to 30.4°C after four times of continuous excitation, indicating that BI NPs had satisfactory photothermal stability. In addition, as shown in Figure 3D, the calculated photothermal conversion efficiency of BI NPs was 36%, comparable to that of the previously reported ICG nanomaterials [47, 48], demonstrating their high potential for photothermal conversion.

### *In vitro* antibacterial research

**Figure 4.**
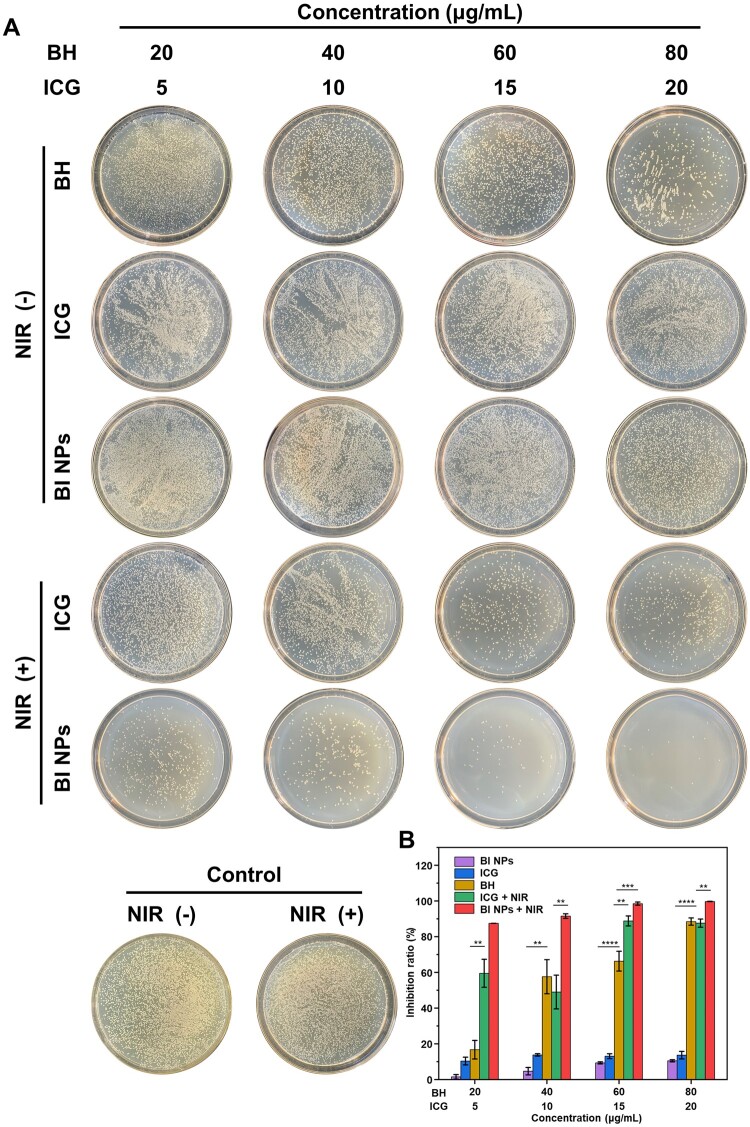
BI NPs-based synergistic antibacterial effect against *S.aureus*. (**A**) Photographs of bacterial colonies after different treatment. (**B**) The corresponding antibacterial efficiency of different treatment, **P < 0.01, ***P < 0.001 and ****P < 0.0001 (n = 3).

Through the plate counting method, the eradication effect of BI NPs on *S.aureus in vitro* was assessed. Figure 4A shows the colonies of *S.aureus* after co-culture with different groups of samples. The inhibition rate was then determined by counting the colonies on each agar plate (Figure 4B). Each treatment group showed a certain degree of bacteriostatic impact compared to the control group. Notably, the BI NPs + NIR group displayed a considerably superior antibacterial activity. Specifically, at the same concentration (25 μg/ml) of BI NPs, the inhibition rate of *S.aureus* was 87.50% with NIR irradiation and 1.54% without NIR irradiation. In addition, the inhibition rate of *S.aureus* was 16.71% in the BH (20 μg/ml) group, 10.38% in the ICG (5 μg/ml) group, and 59.5% in the ICG + NIR group. Although different treatment groups inhibited bacterial growth in a concentration-dependent manner, the BI NPs + NIR group continued to display the highest inhibition rate among the other groups.

In addition, we also explored the eradication effect of BI NPs on *E.coli* (see Supplementary Figure S4). BI NPs alone had no obvious antibacterial effect on *E.coli*. In contrast, the BI NPs + NIR group significantly improved the removal effect of *E.coli*. It was worth noting that compared with the antibacterial effect of BI NPs on *S.aureus* and *E.coli*, the inhibition ratio of BI NPs + NIR group on *E.coli* was 84.74% at a concentration of 100 μg/ml, while the inhibition rate on *S.aureus* was 87.5% at a concentration of only 25 μg/ml. This phenomenon was consistent with previous reports that the inhibitory effect of BH on *E.coli* was much lower than that of *S.aureus* [15]. In conclusion, the formation of BI NPs self-assemblies significantly enhanced the broad-spectrum antibacterial capability of eradicating bacteria, and the NIR laser was an essential condition for the self-assembled system to exert antibacterial effects [49, 50]. Previous studies have reported that *S.aureus* was the most common pathogen in skin infections [51], so we selected it as the model bacteria for subsequent experiments to further explore the antibacterial effect of BI NPs.

**Figure 5.**
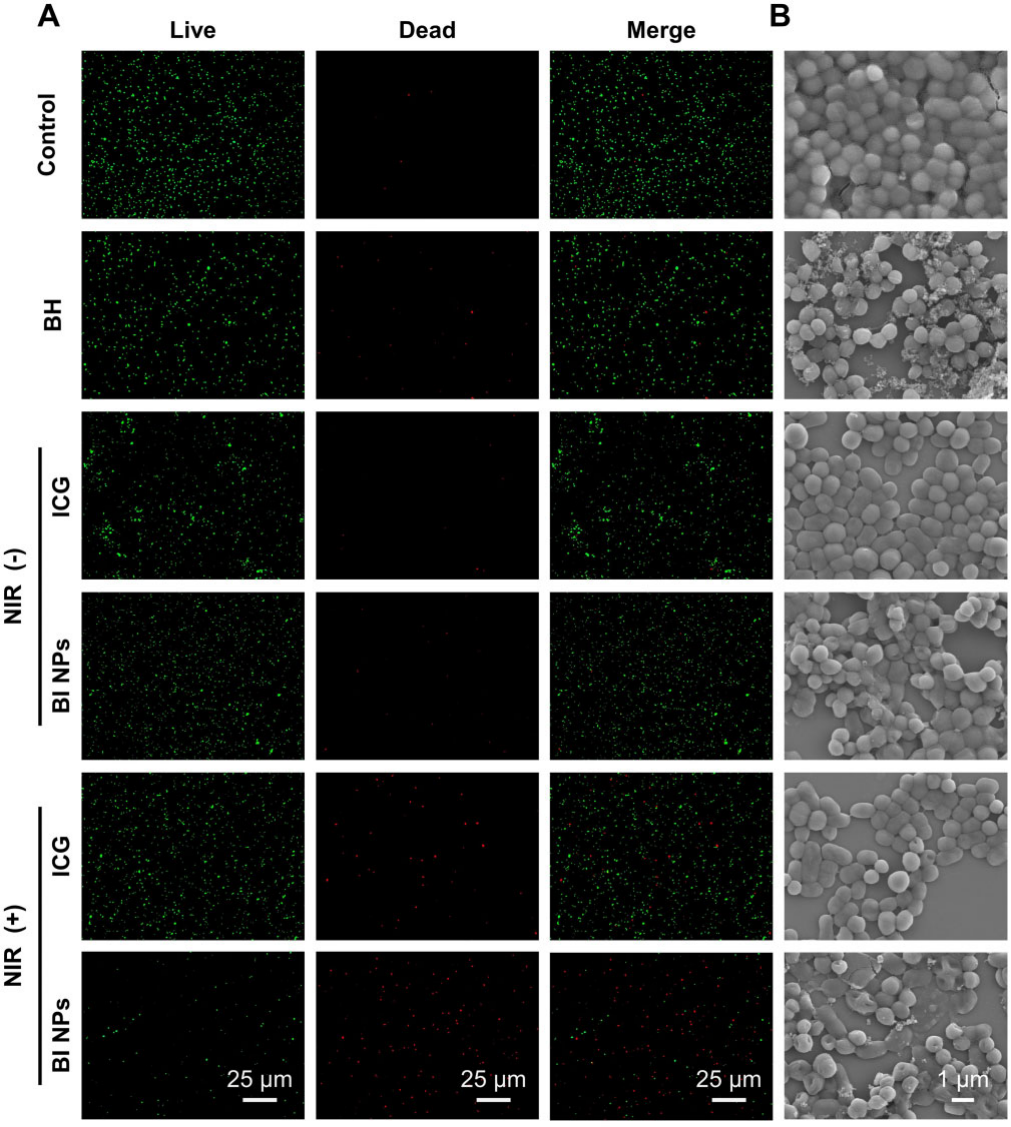
Morphological characterization of *S.aureus*. (A) Fluorescence images and (B) FE-SEM photographs of *S.aureus* treated by BH, ICG, BI NPs, ICG + NIR and BI NPs + NIR.

To further explore the eradication effect of BI NPs on *S.aureus*, the activity and morphological changes of bacteria were observed by fluorescence microscopy and FE-SEM observations, respectively. The microscopic fluorescence results illustrated the chemotherapy-PTT bacterial clearance effect, as shown in Figure 5A. It was observed that there was only a tiny amount of dead bacterial cells in the control group. In contrast, after treatment with BH, ICG, ICG + NIR, and BI NPs, the number of dead bacterial cells increased somewhat, but most bacterial cells still survived. However, most bacterial cells died, and only a few survived in the BI NPs + NIR group. According to Figure 5B, the FE-SEM photographs of the treated bacterial cells indicated that normal *S.aureus* cells were spherical with a smoothing surface, presenting adhesion between bacterial cells. After BH, ICG, ICG + NIR and BI NPs treatment, the results indicated a decreased adhesion between bacteria, with wrinkles and ruptures on some bacterial surfaces, indicating that these treatment groups exhibited limited effects on *S.aureus*. Nevertheless, numerous *S.aureus* in the BI NPs + NIR group presented surface depression and rupture, validating the results of fluorescent staining; namely, the BI NPs + NIR group exhibited a noticeable bacteriostatic effect. Under NIR laser irradiation, ICG in BI NPs converted light energy into heat energy. The increase in temperature caused a certain degree of depression and deformation on the surface of bacteria. It promoted the penetration of released BH, which was the possible antibacterial mechanism of BI NPs.

### Evaluation of wound healing *in vivo*

**Figure 6.**
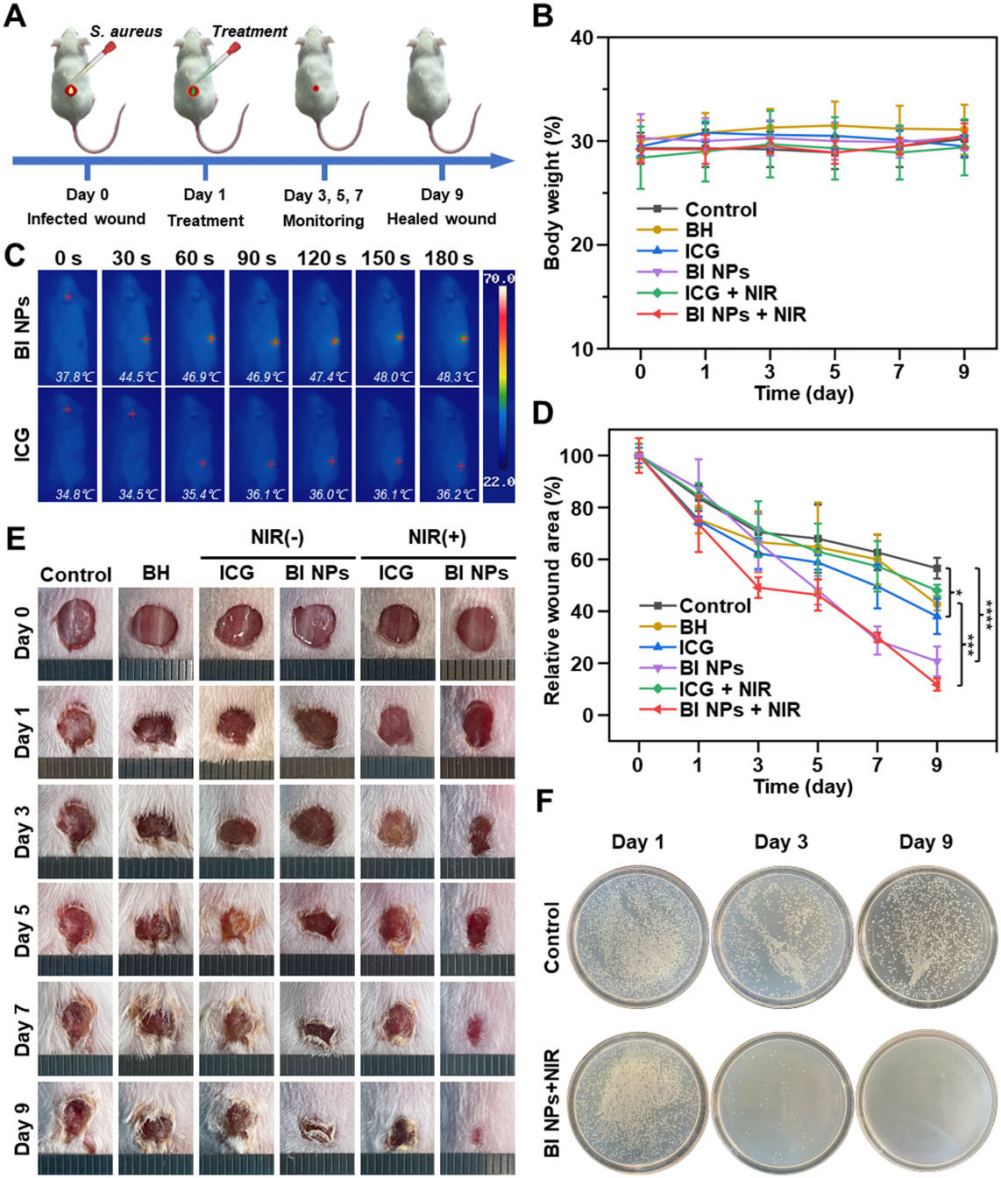
Characterization of antibacterial performance and effect of promoting wound healing. (**A**) Scheme of the therapeutic process in mice. (**B**) Weight change curves of mice during treatment. (**C**) The photothermal images of wounds in the back of mice and (**D**) the quantitative data of wound area, *P < 0.05, ***P < 0.001, and ****P < 0.0001 (n = 3). (**E**) The representative pictures of the wound sites. (**F**) Photographs of cultured colonies isolated from infected wounds on the back of mice on day-1, day-3, and day-9.

The synergistic anti-bacterial effect of BI NPs *in vivo* in mice was further explored by establishing a skin *S.aureus*-infected wound model. Figure 6A showed the primary experimental steps. Firstly, dorsal skin wound was formed on the back of each mouse. After that, it was exposed to *S.aureus* for 24 h to complete the creation of a skin *S.aureus*-infected wound model. On the first day, various groups of samples were applied to the infected wounds of the mice, and then the wound healing process was monitored. According to Figure 6B, there was no discernible variation in the mice weights of whole groups during the course of treatment. To estimate the photothermal conversion effect of BI NPs *in vivo*, the same concentration of ICG was used as a control and the wound sites were irradiated with NIR for 3 min. The infrared imaging camera was used to determine the temperatures during the irradiation process. The photo was recorded every 30 s (Figure 6C). In the BI NPs group, the temperature of the wound area increased by 10.5°C within 3 min. In comparison, the ICG group's wound area temperature barely rose by 1.4°C, with significant differences. These results indicated that the formation of BI NPs self-assemblies helped improve the stability of ICG and achieve an excellent photothermal conversion effect in mice, providing the possibility to eradicate bacteria on the wound sites effectively.

The wound healing of each group was analyzed according to the alterations in wound area (Figure 6D) and wound healing photos (Figure 6E). Compared to each individual therapy group, the synergistic treatment group's capacity for wound healing was dramatically increased. Notably, the BI NPs + NIR group showed satisfactory wound healing performance, with a relative wound area of 11.74% on the ninth day. By comparison, the control group showed a relative wound area of 56.56%, the BH group of 42.71%, the ICG group of 38.02%, the BI NPs group of 20.69% and the ICG + NIR group of 48.04%, indicating that BI NPs combined with NIR laser significantly accelerated the scar formation and overall healing process of wound tissue in mice. Furthermore, comparing the results of *in vitro* and *in vivo* experiments, it was found that the antibacterial effect of BH *in vitro* was superior to BI NPs. In contrast, the antibacterial effect of BI NPs *in vivo* was superior to BH. We believed that due to the hydrophobicity of BH, it would be rapidly cleared by metabolism when used *in vivo*, thus limiting its effect. At the same time, BI NPs achieved a sustained and slow release of BH, extending the action time of BH *in vivo*, and thus improving the antibacterial effect of BH *in vivo*.

By culturing and coating the bacteria at the wound site of the mouse, bacterial growth at the wound site could be observed. Bacteria were sampled at the wound sites every 2 days, cultured on broth agar plates, and photographed. Figure 6F showed photographs of the colonies on the first, third and ninth days, and the rest of photographs could be found in Supplementary Figure S5. On the first day, there were a large number of bacteria at the wound site of each group, indicating that the wound model of *S.aureus* infection was successfully constructed. Then, each group was treated with different samples. On the third day, the number of bacteria in each single treatment group considerably dropped compared to the control group. In addition, the BI NPs + NIR group almost eliminated the bacteria in all wound sites and remained in this state until the ninth day. These findings indicated excellent sustained anti-bacterial ability could be due to the slow release of BH in BI NPs.

**Figure 7.**
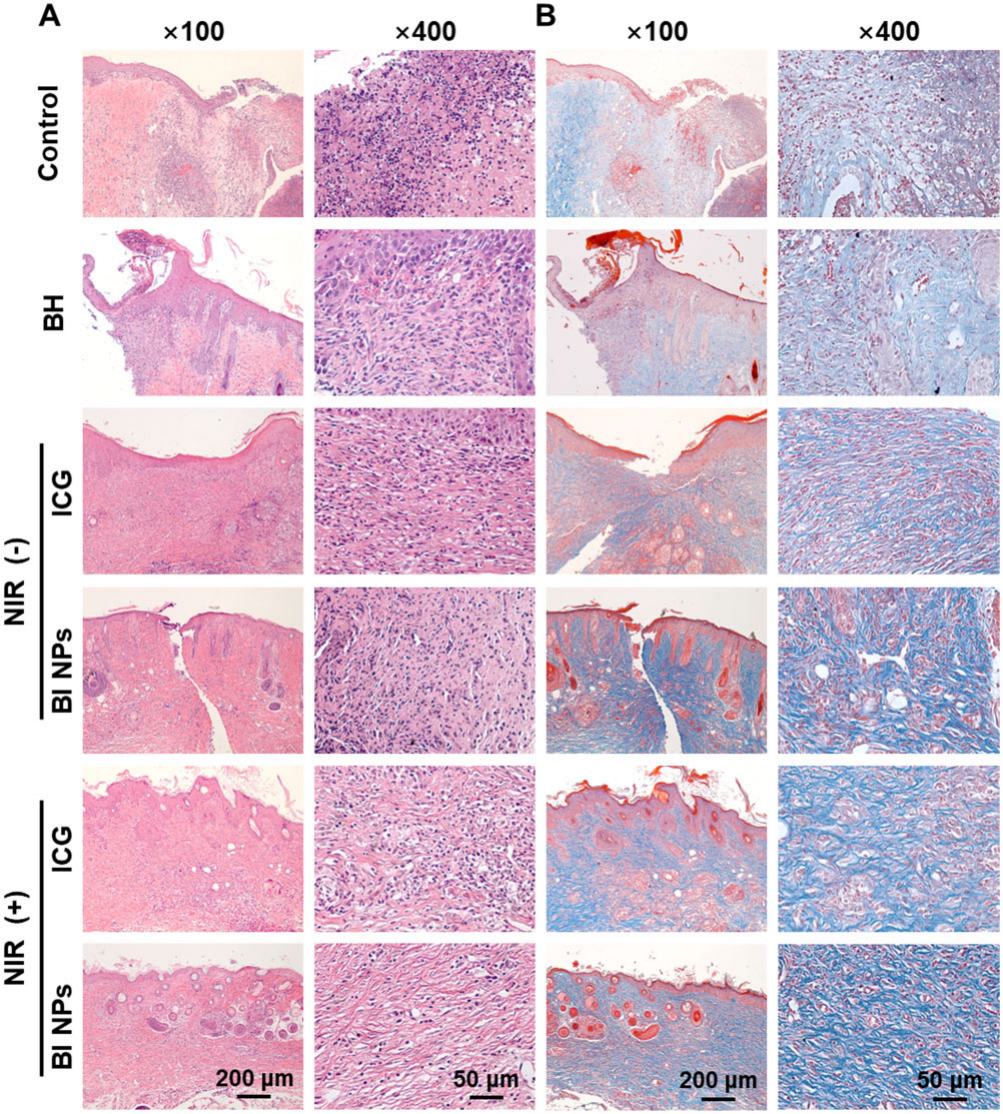
Micrographs of wounds after 9 days of treatment by BH, ICG, BI NPs, ICG + NIR and BI NPs + NIR. (**A**) H&E staining and (**B**) Masson’s trichrome staining of regenerated tissues from the wound site.

On the ninth day, the healing efficiency of wounded tissues was further evaluated using H&E staining and Masson’s trichrome staining [52]. When the body was infected with bacteria, inflammatory cells accumulate at the site of infection. The photographs of H&E staining showed that the control group still included a significant number of inflammatory cells, denoting severe bacterial infection (Figure 7A). Each treatment group showed some reduction in inflammatory cell infiltration as compared to the control group. Specifically, BI NPs + NIR group showed fewer inflammatory cells, showing excellent synergistic antibacterial effects *in vivo*. According to the principle of Masson’s trichrome staining, muscle fibers are red, collagen fibers are blue, and the nucleus is black. Indeed, collagen deposition is a crucial feature of wound healing [53]. The results of Masson’s trichrome staining were shown in Figure 7B, revealing that the BI NPs + NIR group showed more collagen deposition and coarser collagen fibers than other treatment groups.

These findings indicated that BI NPs possessed the potential to promote wound healing, which might be attributed to their effective drug release and synergistic antimicrobial strategy. *S.aureus* adversely affected wound healing by secreting virulence factors and inhibiting fibroblast proliferation and differentiation [54]. NIR-induced PTT could only kill bacteria quickly but possessed no long-term antibacterial ability [55]. BI NPs not only exerted the antibacterial effect of PTT during NIR laser irradiation but also triggered the sustained release of BH for long-lasting bacterial inhibition, thus eliminating the adverse effect of *S.aureus* on the wound healing process. In addition, mild heat could promote cell proliferation and differentiation, collagen deposition and capillaries regeneration, thereby accelerating wound healing [56, 57]. In conclusion, BI NPs combined with NIR laser achieved sustained bacterial removal, accelerated wound healing, and reduced the possibility of infection recurrence.

### Biocompatibility

**Figure 8.**
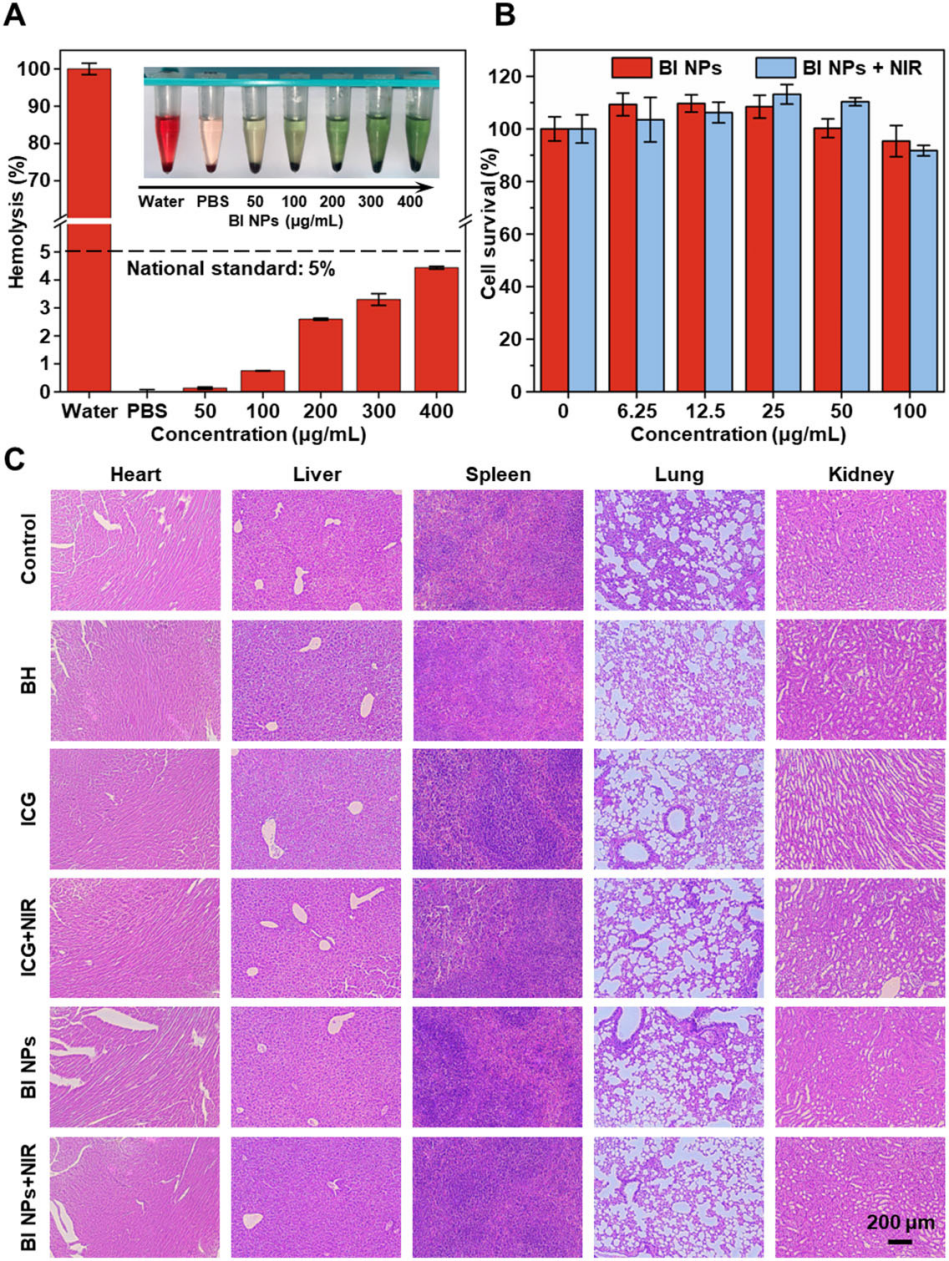
The biocompatibility of BI NPs. (**A**) Hemolysis ratio and related images of whole blood after administering of various BI NPs concentrations. (**B**) Cell viability of L929 cells after 24 h of treatment with multiple doses of BI NPs with or without NIR. (**C**) Significant organs after 9 days of treatment were stained with H&E staining.

The biocompatibility of BI NPs was investigated by blood compatibility and cell compatibility. Figure 8A showed the hemolysis rate of BI NPs at different concentrations with or without NIR. The hemolysis rate was below 5% (4.78%) when the concentration of BI NPs was increased to 400 μg/ml, which was within the acceptable limit of ASTM E2524 08 standard. Furthermore, the cytocompatibility of BI NPs was evaluated by the CCK-8 kit. Equal number of L929 cells were treated separately with different concentrations of BI NPs dispersion with or without NIR for 24 h. The cell viability was calculated and was shown in Figure 8B. The findings demonstrated that the cell viability of each treatment group was more than 80%, confirming that BI NPs with or without NIR had low cytotoxicity to L929 cells in this range and showed excellent compatibility with cells *in vitro*. In addition, no significant histological abnormalities or organ damage were detected H&E-stained major organs (Figure 8C). According to these findings, BI NPs demonstrated excellent biocompatibility *in vivo* and could be employed as a desirable antibacterial modality for synergistic antibacterial therapy.

## Conclusion

In summary, an NIR-responsive nanoplatform (BI NPs) was proposed for chemotherapy/PTT synergistic therapy and repairment of *S.aureus*-infected wounds. BI NPs were prepared by a facile self-assembly approach, indicating high stability, excellent biocompatibility and effective photothermal performance. The carrier-free BI NPs achieved effective and accurate BH release (67.73%) in response to the NIR laser. At the same time, BI NPs showed enhanced synergistic antibacterial activities *in vitro* compared to a single medication or PTT. Furthermore, *in vivo,* experiments demonstrated that BI NPs combined with NIR laser achieved long-term bacterial removal, accelerated wound healing, and reduced the risk of infection recurrence. In conclusion, this NIR-responsive nanoplatform was expected to provide a feasible approach for the repairment of infected wound.

## Supplementary Material

rbad076_Supplementary_Data
