# Supplementary material for: NIR-responsive carrier-free nanoparticles based on berberine hydrochloride and indocyanine green for synergistic antibacterial therapy and promoting infected wound healing
Source: Regen Biomater. 2023 Aug 30;10:rbad076. doi: 10.1093/rb/rbad076 (PMC10558098; doi:10.1093/rb/rbad076)
Supplement: rbad076_Supplementary_Data [file rbad076_supplementary_data.docx]

Supplementary data

**NIR-responsive Carrier-free Nanoparticles based on Berberine Hydrochloride and Indocyanine Green for Synergistic Antibacterial Therapy and Promoting Infected Wound Healing**

**You-Yu Duan^1,2^, Pei-Yao Xu^1,2^, Pan-Yuan Ge^1,2^, Lin-Fei Chen^1,2^, Ying Chen^1,2^, Ranjith Kumar Kankala^1,2^, Shi-Bin Wang^1,2^, Ai-Zheng Chen^1,2,*^**

^1^Institute of Biomaterials and Tissue Engineering, Huaqiao University, Xiamen, Fujian 361021, PR China.

^2^Fujian Provincial Key Laboratory of Biochemical Technology, Huaqiao University, Xiamen, Fujian 361021, PR China.

*Correspondence address. Email: [azchen@hqu.edu.cn](mailto:azchen@hqu.edu.cn) (A. Z. C.)

1. **Experimental Details**

**1.1 Calculation of the cumulative release of BH from BI NPs**

The cumulative drug release amount at time n (Mn) was calculated as:

$$M_{n}=C_{n}\times V+\sum C_{n-1}V_{s}$$

Where $C_{n}$ is the concentration of BH in the release medium at time n, V is the total volume of the release medium and $V_{s}$ is the volume of each sample that was collected for measurement. Finally, the cumulative BH release ratio was calculated following the formula:

$$Cumulative BH release \left( \% \right)=\frac{M_{n}}{M_{\mathrm{total}}}\times100$$

Where $M_{\mathrm{total}}$ is the total amount of BH encapsulated in the BI NPs.”

**1.2 Calculation of the photothermal conversion efficiency**

The calculation process of photothermal conversion efficiency (η) was as follows:

$$\eta=\frac{\mathrm{hS}\left( T_{\max}-T_{\mathrm{surr}} \right)-Q_{\mathrm{Dis}}}{I(1-{10}^{-A_{808}})}$$

Where h represents the heat transfer coefficient; S represents the surface area of the container; T_max_ and T_surr,_ respectively, represent maximum steady-state temperature and room temperature; Q_Dis_ represents the heat dissipation of the laser under the action of solvent and container; I represents the laser power, and A_808_ represents the absorbance of the sample at 808 nm.

hS is calculated referring to the following equation:

$$hS=\frac{mC_{\mathrm{water}}}{\tau_{s}}$$

Where m represents the quality of the sample; τ_s_ represents the related time constant; C represents the specific heat capacity of solution (C_water_ = 4.2 J/(g·℃)).

$$\tau_{s}=\frac{t}{-ln \theta}$$

Where θ represents a dimensionless parameter called driving force temperature.

$$\theta=\frac{T-T_{\mathrm{surr}}}{T_{\max}-T_{\mathrm{surr}}}$$

Where T denotes the temperature of the BI NPs dispersion measured during the irradiation process.

1. **Results**


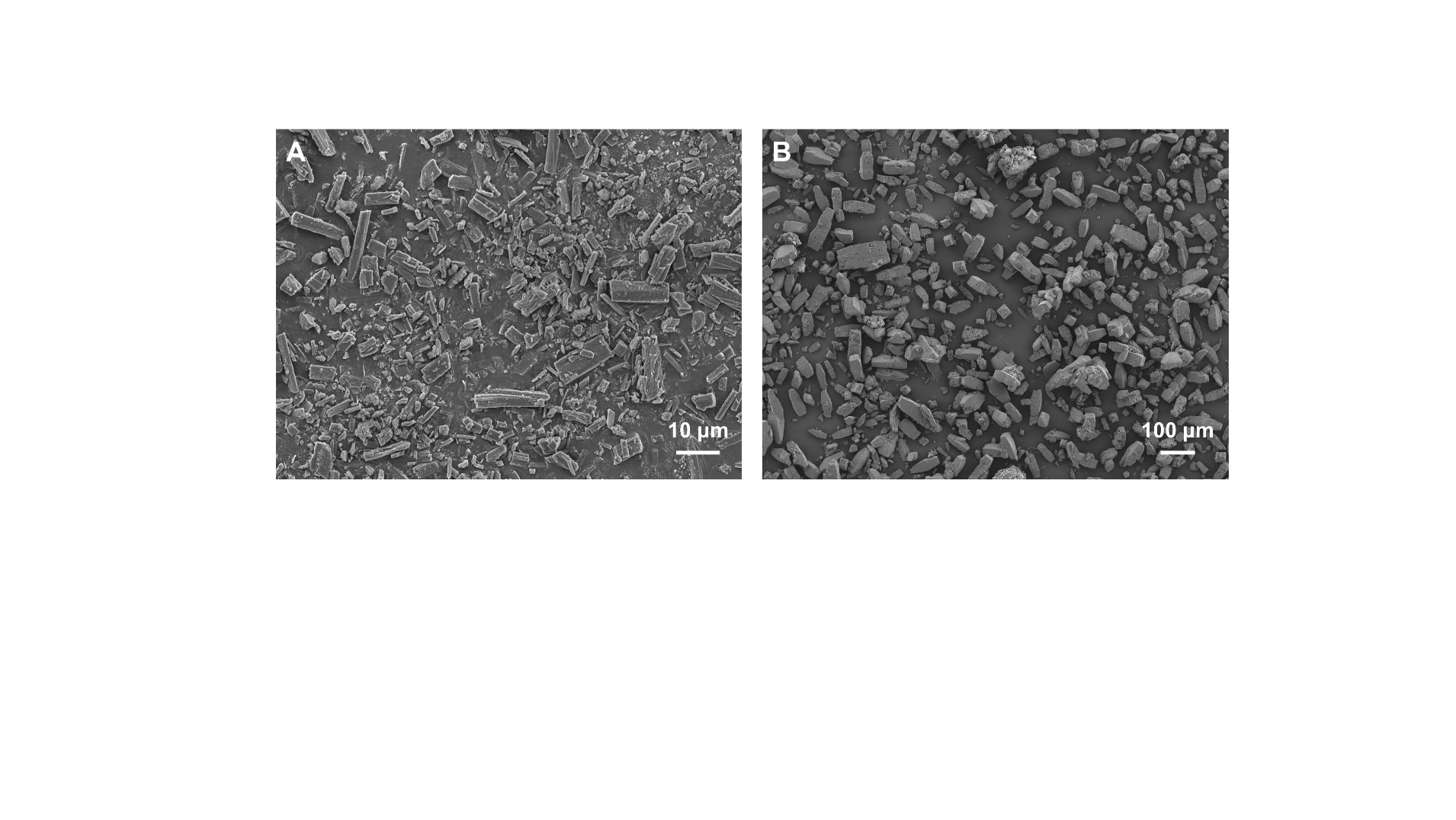


**Fig. S1** SEM photographs of unprocessed drugs. **(A)** BH, **(B)** ICG.


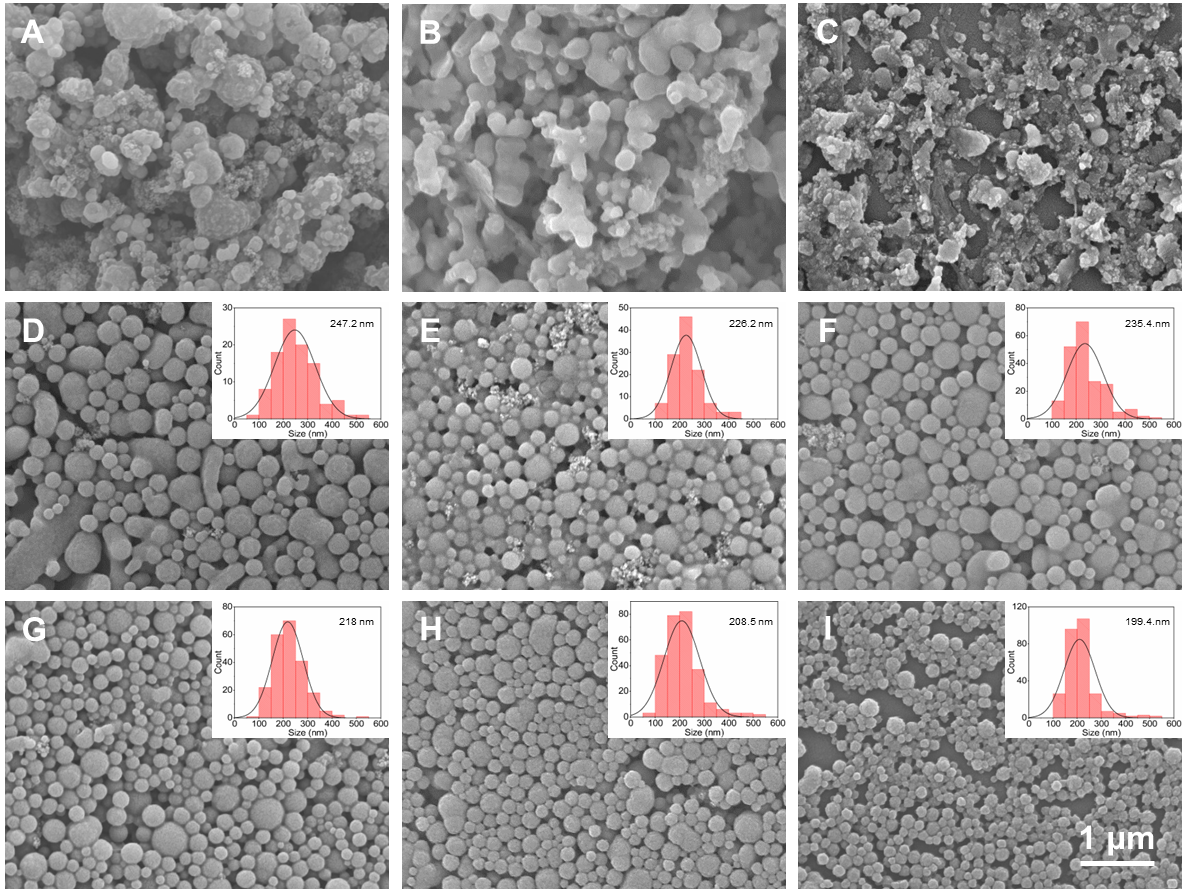


**Fig. S2** SEM photographs of BH-ICG nanoassemblies assembled from different BH-ICG mass ratios. (**A**)1: 0.25, (**B**)1: 0.5, (**C**)1: 0.6, (**D**)1: 0.7, (**E**)1: 0.8, (**F**)1: 0.9, (**G**)1: 1, (**H**)1: 2, (**I**)1: 4.


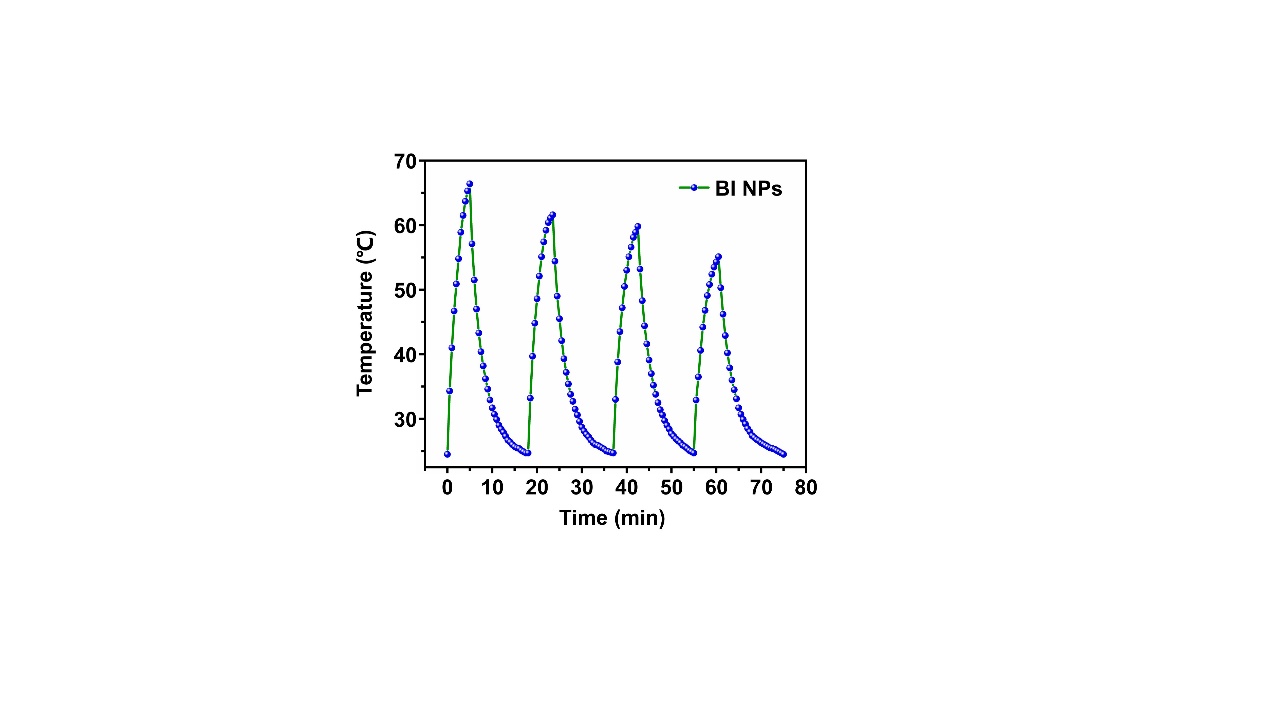


**Fig. S3** Photothermal stability study of BI NPs during four circles of heating-cooling processes.


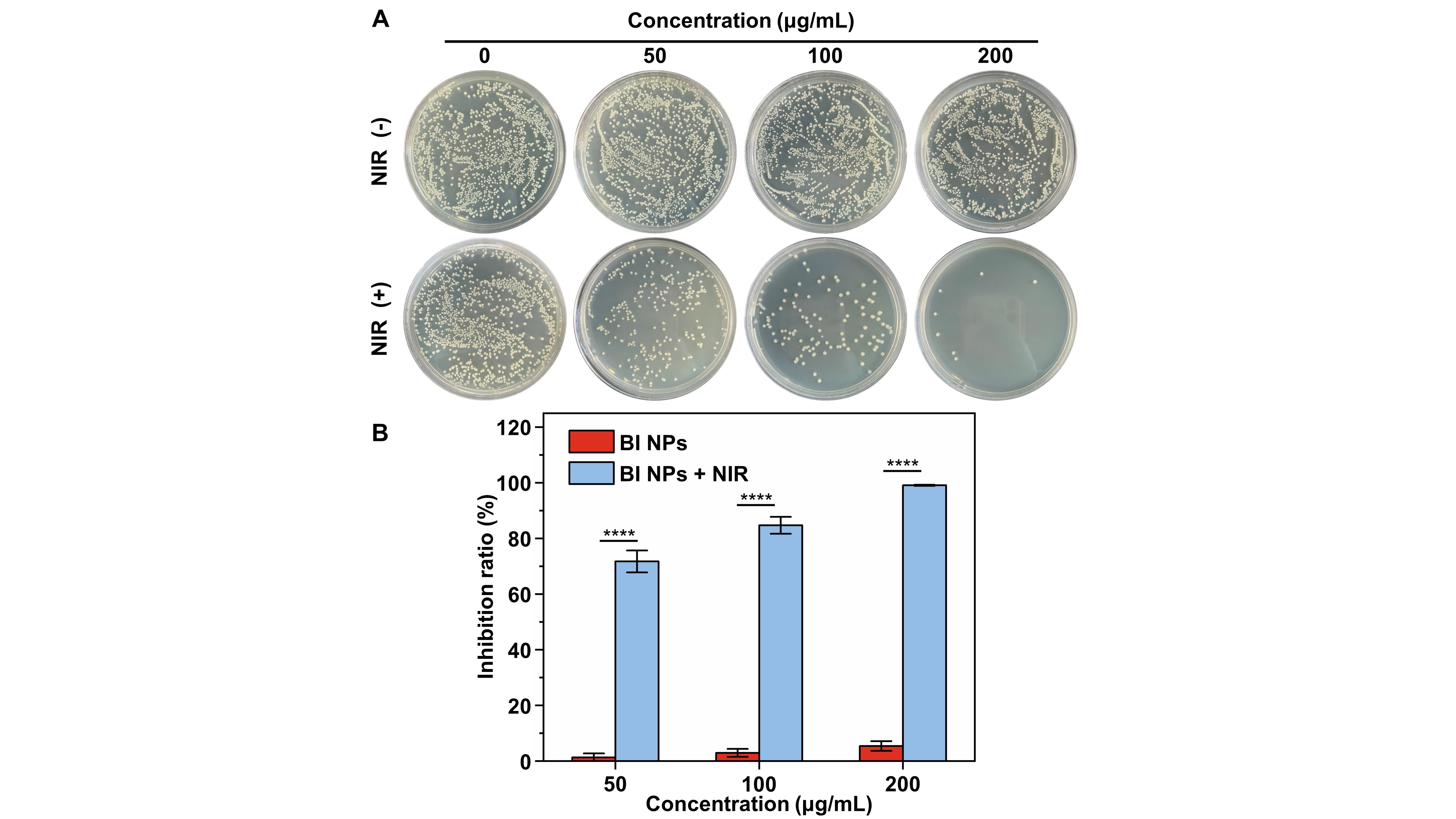


**Fig. S4** BI NPs based synergistic antibacterial effect against *E. coli*. (A) Photographs of bacterial colonies after different treatment. (B) The corresponding antibacterial efficiency of different treatment, *****p < 0.0001* (n = 3).


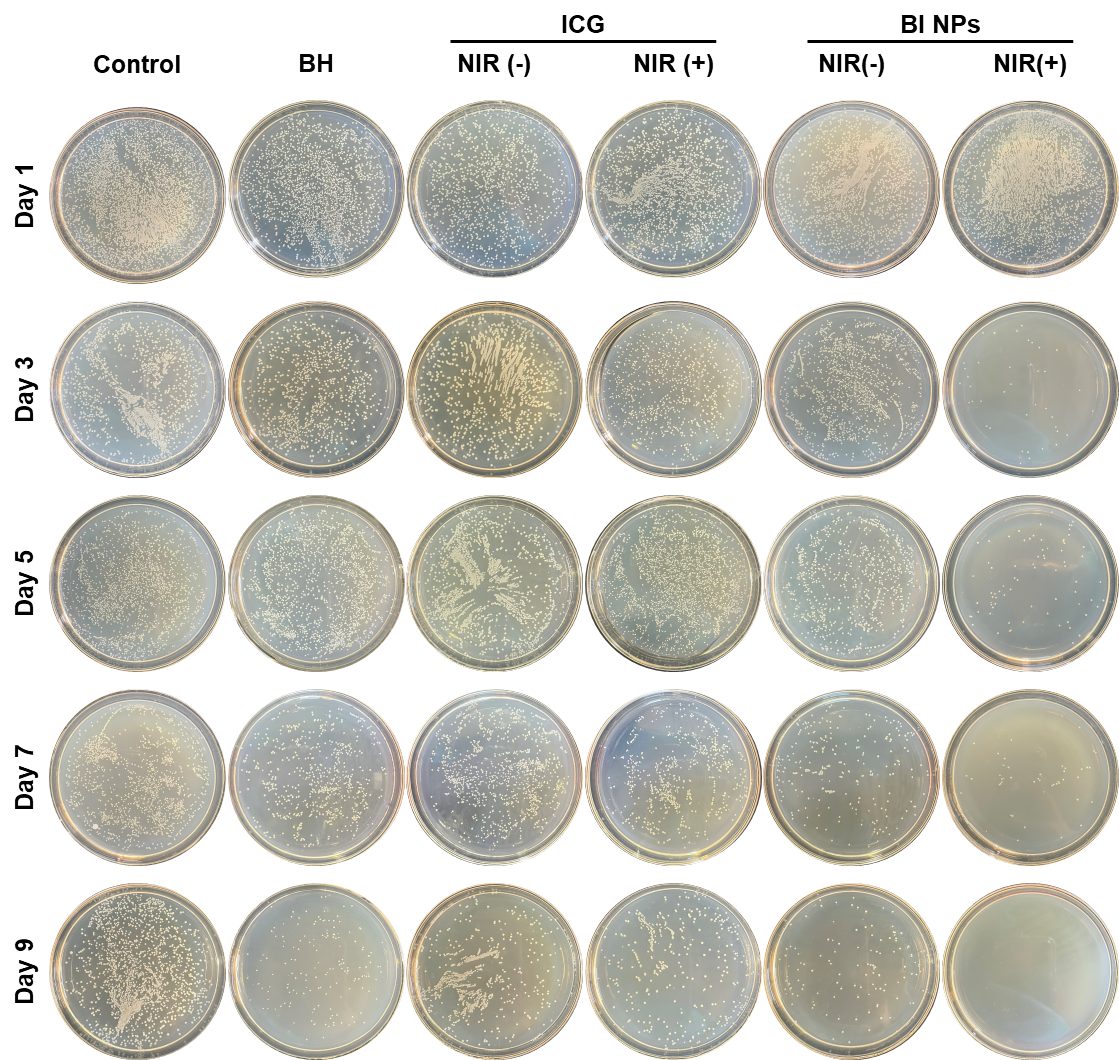


**Fig. S5** The photographs of *S. aureus* colonies isolated from the wound site grown on broth agar plates after receiving various treatments on days 1-9.
